# Prevalence of metal hypersensitivity in pediatric spine surgery

**DOI:** 10.1007/s43390-024-01030-7

**Published:** 2025-01-14

**Authors:** A. Scott Emmert, Tiffany Ruan, Michael G. Sherenian, Amal H. Assa’ad, Nichole Leitsinger, Lindsay Schultz, Viral V. Jain, Peter F. Sturm, Alvin C. Jones

**Affiliations:** 1 Division of Orthopaedic Surgery, Cincinnati Children’s Hospital Medical Center, 3333 Burnet Ave, Cincinnati, OH 45242 USA; 2 Division of Allergy and Immunology, Cincinnati Children’s Hospital Medical Center, Cincinnati, OH USA

**Keywords:** Metal, Hypersensitivity, Pediatric, Spine, Scoliosis

## Abstract

**Purpose:**

Delayed metal hypersensitivity reactions can cause complications in spine surgery. Currently, there is no information on the prevalence of metal hypersensitivity in pediatric patients undergoing spine surgery. The objective of this study is to determine the prevalence of metal hypersensitivity in pediatric patients undergoing spinal instrumentation.

**Methods:**

Retrospective chart review of patients who underwent spinal instrumentation with or without fusion at a single institution, from January 1, 2014, to December 31, 2020, was performed. Patients were pre-screened for history of allergic diseases, including previous reaction to metals, prior to surgery. Patch metal allergy testing (PMAT) for metal hypersensitivity was also performed.

**Results:**

Of the 796 pediatric patients who underwent spinal instrumentation procedures from 2014 to 2020, 118 (15%) screened positive for metal hypersensitivity. However, the number of patients with documented evidence of metal hypersensitivity diminished to 26 (3%) after PMAT verification. Nickel hypersensitivity was most prevalent, with 20 patients (16.9% of positive screening; 2.5% of all instrumented patients) demonstrating positive skin patch tests. The other most prevalent metal hypersensitivities included cobalt in 9 patients (7.6%; 1.1%), manganese in 3 patients (2.5%; 0.4%), and copper in 1 patient (0.8%; 0.1%). with a number needed to treat (NNT) of 5.

**Conclusions:**

This study suggests that routine pre-operative PMAT is not necessary in all pediatric spine patients yet should be considered if patients report a history of prior metal hypersensitivity reactions.

## Introduction

Metal implants are commonplace in orthopaedic surgery. For fracture management, titanium alloy or stainless-steel implants are frequently used. Also, during spinal fusion and instrumentation procedures, titanium, cobalt chromium, and stainless-steel rods and screws are most often used [1]. Within these implants exist varying levels of metallic elements. Some of these elements, such as nickel, have been known to have a high prevalence of hypersensitivity reactions in the general population. Metal hypersensitivity is problematic in the orthopaedic population because it can lead to dermatitis reactions, delayed wound healing, recurrent pain, swelling, erythema, and osteolysis [2, 3].

Controversy exists on whether to test patients pre-operatively before receiving orthopaedic implants. Currently, most of the research on this topic has been in patients receiving total joint arthroplasties. Recent literature supports not requiring all patients to undergo pre-operative testing. Instead, it recommends surgeons communicate with their patients and if there is a concern, then testing should be performed [4, 5].

Several reports describe metal hypersensitivity reactions in adult spine patients [6–8]. However, in pediatric spinal surgery the literature is significantly lacking, with only one apparent case-report [9]. Adolescent idiopathic scoliosis is a common disease with a prevalence falling somewhere between 0.5–5%, and about a tenth of patients require surgery [10]. Information on the prevalence of metal hypersensitivity in this population is currently unavailable. This study attempts to quantify the prevalence of metal hypersensitivity in a pediatric patient population undergoing spinal instrumentation.

## Materials and methods

This is a retrospective cohort study investigating metal hypersensitivity in all patients under 18 years old, having posterior spinal fusion with instrumentation at a single pediatric academic medical center from January 1, 2014, to December 31, 2020. Several weeks before spinal fusion with instrumentation all patients at this institution undergo metal hypersensitivity screening with a pre-operative question, “Has the patient ever reacted with a red patchy rash to contact with metal buckles, snaps, zippers, or cheap jewelry?” Patients with positive responses are referred for patch metal allergy testing (PMAT). PMAT is performed and interpreted by board-certified Allergy and Immunology specialists. The PMAT tests include nickel sulfate hexahydrate 5%, cobalt(II) sulfate 2.5%, manganese chloride 0.5%, copper sulfate hexahydrate 2%, aluminum hydroxide 10%, titanium(IV) oxide 0.1%, ammonium hepatomolybdate 1%, vanadium pentoxide 10%, and chromium(III) chloride 1%. A positive skin patch test on PMAT is defined as a skin hypersensitivity reaction of 2+. If a metal hypersensitivity is found, then the implant for the procedure is chosen to appropriately avoid the metal element to which the patient has hypersensitivity.

The electronic medical record was used to obtain pertinent patient data including PMAT results. Only patients undergoing elective spinal fusion and instrumentation were included in this study. Any patients undergoing multiple procedures were only included once in the analysis, with the original procedure being included. If patients did not undergo instrumentation for their spinal procedure, they were excluded from the analysis. Also, patients that underwent emergency or non-elective spinal instrumentation and fusion procedures were excluded. PMAT results were analyzed by type of metal eliciting hypersensitivity. Metals were obtained in tubes of the metal in petrolatum from Allergeaze^®^, Alberta, Canada and placed on Finn Chambers on scanpore® from SmartPractice, Phoenix, Arizona (Table 1).

**Table 1:** Composition of metal alloys in standard surgical implants of cobalt chrome, titanium, and stainless steel. Adapted from the American Society for Testing and Materials (ASTM) Medical Device Standards and Implant Standards [11–13]

Element	Cobalt chrome (%)	Titanium (%)	Stainless steel (%)
Chromium	26–30		17–19
Molybdenum	5–7		2.2–3
Nickel	< 1		13–15
Silicone	> 1		< 0.75
Iron	< 0.75	< 0.25	Balance
Carbon	< 0.35	< 0.08	< 0.5
Manganese	< 1		< 2
Phosphorus			< 0.5
Copper			< 0.5
Sulfur			< 0.5
Nitrogen			< 0.5
Cobalt	Balance		
Aluminum		5.5–6.5	
Vanadium		3.5–4.5	
Oxygen		< 0.13	
Hydrogen		< 0.012	
Titanium		Balance	

The prevalence of metal hypersensitivity was evaluated in the context of both positive and negative pre-operative screening results. Number needed to treat (NNT) was calculated to evaluate the diagnostic utility of history and laboratory data compared to PMAT results in assessing metal hypersensitivity. The number of patients who screened positive for a history of metal hypersensitivity with positive PMAT tests compared to all patients who received spinal instrumentation were analyzed with GraphPad Prism (GraphPad Software, San Diego, California) to calculate the NNT. This study received Institutional Review Board approval and exemption to report this data without consent.

## Results

**Fig. 1:**
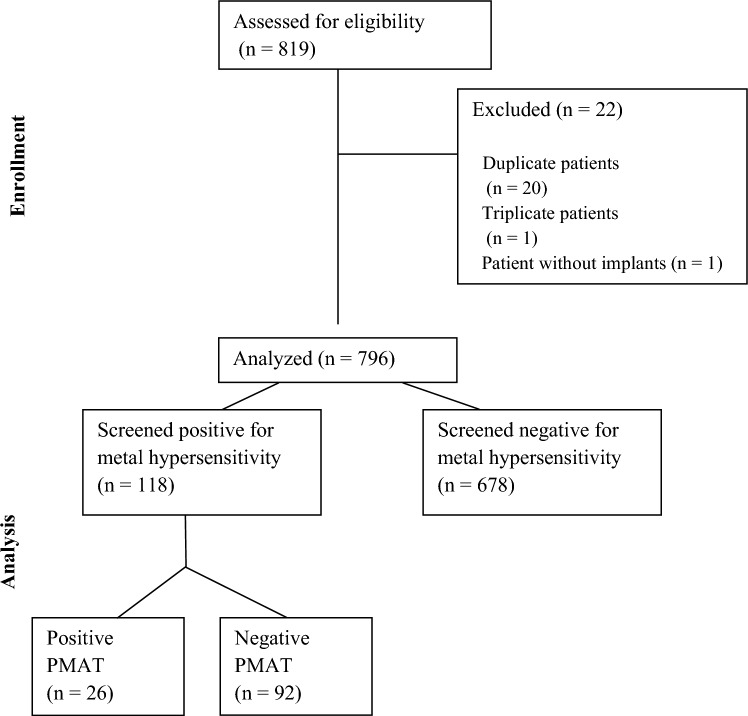
CONSORT 2010 flow diagram of the algorithm for enrollment and analysis of patients undergoing pediatric spine surgery at Cincinnati Children’s Hospital Medical Center from 2014–2020

A schematic representation of all patients fulfilling study criteria is shown in Fig. 1. Prior to application of exclusion criteria, 819 pediatric patients underwent orthopaedic spinal surgeries during the period from January 1, 2014, to December 31, 2020. Of these, 796 patients received spinal instrumentation. Patients undergoing redundant surgeries (*n* = 20 duplicate; *n* = 1 triplicate) or who did not receive implants (*n* = 1) were excluded. Among patients receiving spinal instrumentation, 118 screened positive for a history of metal hypersensitivity on the pre-operative screening questionnaire while 678 screened negative. Of the patients who screened positive and were referred for PMAT, 26 exhibited a positive skin patch test while 92 exhibited a negative skin patch test. The number needed to treat (NNT) for PMAT testing among patients who screened positive for a history of metal hypersensitivity compared to all patients who received spinal instrumentation was 5.

### Patch metal allergy testing

Metal-specific sensitivities to skin patch testing for the 118 patients who screened positive on the pre-operative questionnaire as well as all patients undergoing spinal instrumentation are shown in Table 2. 26 patients (22.0% of positive screening; 3.3% of all instrumented patients) demonstrated hypersensitivity upon skin patch testing to any of the nine metals utilized. Nickel hypersensitivity was most prevalent, with 20 patients (16.9% of positive screening; 2.5% of all instrumented patients) demonstrating positive skin patch tests to nickel sulfate hexahydrate 5%. The other most prevalent metal hypersensitivities included cobalt in 9 patients (7.6% of positive screening; 1.1% of all instrumented patients), manganese in 3 patients (2.5% of positive screening; 0.4% of all instrumented patients), and copper in 1 patient (0.8% of positive screening; 0.1% of all instrumented patients). No patients demonstrated hypersensitivities to aluminum, titanium, ammonium, vanadium, or chromium.

**Table 2:** Patch metal allergy testing (PMAT) results of pediatric orthopaedic spinal instrumentation patients who screened positive for history of previous reaction to metals

Variable	Screened positive (*n* = 118)
Positive patch test, *n* (% positives)	26 (22.0%)
Nickel sulfate hexahydrate 5%	20 (16.9%)
Cobalt(II) sulfate 2.5%	9 (7.6%)
Manganese chloride 0.5%	3 (2.5%)
Copper sulfate hexahydrate 2%	1 (0.8%)
Aluminum hydroxide 10%	0
Titanium(IV) oxide 0.1%	0
Ammonium hepatomolybdate 1%	0
Vanadium pentoxide 10%	0
Chromium(III) chloride 1%	0

## Discussion

This study found an overall low rate of metal hypersensitivity in our pediatric spinal instrumentation population. Similar to previous studies, nickel sulfate was revealed to be the most common metal hypersensitivity [3, 14, 15]. It has been estimated that up to 17% of women and 3% of men have hypersensitivity to nickel, and suggested that the prevalence increases with repeated exposure [16]. Nickel is a common component in metallic jewelry often used by children and frequently found in the environment. Furthermore, evidence has shown that pierced ears and the use of these types of jewelry is a significant risk factor for nickel allergy [15]. Therefore, some speculate that the sensitization to nickel would increase with age and frequency of exposure. Unfortunately, this would only explain the high prevalence of nickel hypersensitivity and not why we found cobalt to be the second-highest hypersensitivity reaction, since cobalt is not frequently encountered in the environment by the public.

If indeed the true prevalence is 3% as we found it would suggest that routine pre-operative PMAT is not necessary in all pediatric spine patients. However, when pre-operative PMAT is combined with a positive result on a preoperative metal hypersensitivity screening question the NNT is 5, which appears to be more useful.

Our findings align with studies of metal hypersensitivity to orthopaedic implants in areas outside of pediatric spinal instrumentation procedures. Atanaskova Mesinkovska et al. demonstrated the effectiveness of skin patch testing on surgical decision-making in patients with suspected metal hypersensitivity related to orthopaedic implants [3]. Furthermore, Kręcisz et al. concluded that patch test screening should be obligatory before othopaedic implantation in patients with a known history of metal dermatitis [17].

This information must be interpreted with the understanding of several assumptions and limitations. First, a screening tool was used to decide which patients underwent skin patch testing. We must acknowledge that this tool was developed by the spine center at one institution and has no external validation. However, we are not aware of any other validated screening tool that serves a similar purpose. Also, some questions remain about the most appropriate pre-operative testing method for metal hypersensitivity and whether PMAT only demonstrates a cutaneous response, which may be different to the response elicited by an indwelling implant. Another potential limitation relates to how accurately allergists or dermatologists carry out patch tests, because factors that can lead to false-negative tests include inadequate percutaneous penetration, technique error, immunosuppressive therapy, and ultraviolet exposure [18]. Other methods, including lymphocyte transformation testing or lymphocyte activation testing, exist to investigate the presence of metal hypersensitivity. Yet, skin patch testing remains the preferred method to use when consulted for preimplant and postimplant metal hypersensitivity reactions [19]. In the joint arthroplasty population, Carossino et al. suggested that the most suitable method for evaluating systemic allergies was both skin patch testing and lymphocyte transformation test [20]. They suggested that combining testing can also help when monitoring the possibility for de novo metal sensitization in patients with implanted metal devices. Nevertheless, it must be stated that there are challenges in obtaining these lymphocyte transformation tests, which include costs and availability. Many of these lymphocyte transformation or activation tests are not covered by insurance, also they are not universally performed in most laboratories in the United States.

A low prevalence of metal hypersensitivity was found in this population of pediatric spine patients undergoing instrumentation and fusion. The NNT of 5 calculated in our study suggests that five pediatric patients undergoing spinal instrumentation would have to undergo PMAT testing for one patient to demonstrate laboratory evidence of metal hypersensitivity. Further, laboratory evidence of metal hypersensitivity upon PMAT does not correlate with post-operative allergic reactions based on preliminary data from our cohort. This low prevalence would suggest that it is unnecessary to test all patients pre-operatively, which is consistent with much of the literature seen in adult joint arthroplasty [21]. Nevertheless, using a pre-operative screening question asking about prior metal sensitivity reactions does appropriately narrow down the population of patients to a reasonable number needed to treat.

## Data Availability

The data is available on request to the corresponding author.

## References

[1] Warburton A, Girdler SJ, Mikhail CM, Ahn A, Cho SK (2020) Biomaterials in spinal implants: a review. Neurospine 17:101–110. 10.14245/ns.1938296.148 31694360 10.14245/ns.1938296.148 PMC7136103

[2] Hallab N, Merritt K, Jacobs JJ (2001) Metal sensitivity in patients with orthopaedic implants. J Bone Jt Surg Am 83:428–436. 10.2106/00004623-200103000-00017 10.2106/00004623-200103000-00017 11263649

[3] AtanaskovaMesinkovska N, Tellez A, Molina L, Honari G, Sood A, Barsoum W, Taylor JS (2012) The effect of patch testing on surgical practices and outcomes in orthopedic patients with metal implants. Arch Dermatol 148:687–693. 10.1001/archdermatol.2011.2561 22351785 10.1001/archdermatol.2011.2561

[4] Razak A, Ebinesan AD, Charalambous CP (2013) Metal allergy screening prior to joint arthroplasty and its influence on implant choice: a delphi consensus study amongst orthopaedic arthroplasty surgeons. Knee Surg Relat Res 25:186–193. 10.5792/ksrr.2013.25.4.186 24368996 10.5792/ksrr.2013.25.4.186 PMC3867611

[5] Wawrzynski J, Gil JA, Goodman AD, Waryasz GR (2017) Hypersensitivity to orthopedic implants: a review of the literature. Rheumatol Ther 4:45–56. 10.1007/s40744-017-0062-6 28364382 10.1007/s40744-017-0062-6 PMC5443731

[6] Goodwin ML, Spiker WR, Brodke DS, Lawrence BD (2018) Failure of facet replacement system with metal-on-metal bearing surface and subsequent discovery of cobalt allergy: report of 2 cases. J Neurosurg Spine 29:81–84. 10.3171/2017.10.SPINE17862 29652237 10.3171/2017.10.SPINE17862

[7] Curley KL, Krishna C, Maiti TK, McClendon J, Bendok BR (2020) Metal hypersensitivity after spinal instrumentation: when to suspect and how to treat. World Neurosurg 139:471–477. 10.1016/j.wneu.2020.04.093 32339728 10.1016/j.wneu.2020.04.093

[8] Kim J (2020) A rare case of delayed hypersensitivity reaction to metal ions secondary to a remnant pedicle screw fragment after spinal arthrodesis. Acta Orthop Traumatol Turc 54:461–464. 10.5152/j.aott.2020.20148 32554366 10.5152/j.aott.2020.20148 PMC7444876

[9] Zielinski J, Lacy TA, Phillips JH (2014) Carbon coated implants as a new solution for metal allergy in early-onset scoliosis: a case report and review of the literature. Spine Deform 2:76–80. 10.1016/j.jspd.2013.09.002 27927446 10.1016/j.jspd.2013.09.002

[10] Konieczny MR, Senyurt H, Krauspe R (2013) Epidemiology of adolescent idiopathic scoliosis. J Child Orthop 7:3–9. 10.1007/s11832-012-0457-4 24432052 10.1007/s11832-012-0457-4 PMC3566258

[11] ASTM International (2023) F75-23 Standard specification for cobalt-28 chromium-6 molybdenum alloy castings and casting alloy for surgical implants (UNS R30075). 10.1520/F0075-23

[12] ASTM International (2023) F1108-21 standard specification for titanium-6aluminum-4vanadium alloy castings for surgical implants (UNS R56406). 10.1520/F1108-21

[13] ASTM International (2017) F745-5 standard specification for 18 chromium-12.5 nickel-2.5 molybdenum stainless steel for cast and solution-annealed surgical implant applications. 10.1520/F0745-95

[14] Frigerio E, Pigatto PD, Guzzi G, Altomare G (2011) Metal sensitivity in patients with orthopaedic implants: a prospective study. Contact Dermatitis 64:273–279. 10.1111/j.1600-0536.2011.01886.x 21480913 10.1111/j.1600-0536.2011.01886.x

[15] Thyssen JP, Linneberg A, Menné T, Johansen JD (2007) The epidemiology of contact allergy in the general population–prevalence and main findings. Contact Dermatitis 57:287–299. 10.1111/j.1600-0536.2007.01220.x 17937743 10.1111/j.1600-0536.2007.01220.x

[16] Thyssen JP, Menné T (2010) Metal allergy–a review on exposures, penetration, genetics, prevalence, and clinical implications. Chem Res Toxicol 23:309–318. 10.1021/tx9002726 19831422 10.1021/tx9002726

[17] Kręcisz B, Kieć-Świerczyńska M, Chomiczewska-Skóra D (2012) Allergy to orthopedic metal implants—a prospective study. Int J Occup Med Environ Health 25:463–469. 10.2478/S13382-012-0029-3 23212287 10.2478/S13382-012-0029-3

[18] Massoumi S, Rizvi Z, Cázares U, Maibach H (2024) Overcoming false-negative patch tests: a systematic literature review. Dermatitis. 10.1089/derm.2023.0065 38181174 10.1089/derm.2023.0065

[19] Schalock PC, Thyssen JP (2013) Metal hypersensitivity reactions to implants. Dermatitis 24:313–320. 10.1097/DER.0b013e3182a67d90 24201465 10.1097/DER.0b013e3182a67d90

[20] Carossino AM, Carulli C, Ciuffi S, Carossino R, ZappoliThyrion GD, Zonefrati R, Innocenti M, Brandi ML (2016) Hypersensitivity reactions to metal implants: laboratory options. BMC Musculoskelet Disord 17:486. 10.1186/s12891-016-1342-y 27881114 10.1186/s12891-016-1342-y PMC5120482

[21] Teo WZW, Schalock PC (2016) Metal hypersensitivity reactions to orthopedic implants. Dermatol Ther (Heidelb) 7:53–64. 10.1007/s13555-016-0162-1 27995484 10.1007/s13555-016-0162-1 PMC5336431

